# Loss of the Arabidopsis Protein Kinases ANPs Affects Root Cell Wall Composition, and Triggers the Cell Wall Damage Syndrome

**DOI:** 10.3389/fpls.2017.02234

**Published:** 2018-01-22

**Authors:** Nora Gigli Bisceglia, Daniel V. Savatin, Felice Cervone, Timo Engelsdorf, Giulia De Lorenzo

**Affiliations:** ^1^Dipartimento di Biologia e Biotecnologie “C. Darwin”, Istituto Pasteur-Fondazione Cenci Bolognetti, Sapienza Università di Roma, Rome, Italy; ^2^Department of Biology, Norwegian University of Science and Technology (NTNU), Trondheim, Norway

**Keywords:** ANPs, MAP kinase kinase kinase, cell wall damage, root swelling, cellulose deficiency, isoxaben

## Abstract

The Arabidopsis NPK1-related Protein kinases ANP1, ANP2 and ANP3 belong to the MAP kinase kinase kinase (MAPKKK) superfamily and were previously described to be crucial for cytokinesis, elicitor-induced immunity and development. Here we investigate the basis of their role in development by using conditional β-estradiol-inducible triple mutants to overcome lethality. In seedlings, lack of ANPs causes root cell bulging, with the transition zone being the most sensitive region. We uncover a role of ANPs in the regulation of cell wall composition and suggest that developmental defects of the triple mutants, observed at the cellular level, might be a consequence of the alterations of the pectic and cellulosic cell wall components. Lack of ANPs also induced a typical cell wall damage syndrome (CWDS) similar to that observed in plants treated with the cellulose biosynthesis inhibitor isoxaben (ISX). Moreover, *anp* double mutants and plants overexpressing single ANPs (*ANP1* or *ANP3*) respectively showed increased and reduced accumulation of jasmonic acid and *PDF1.2* transcripts upon ISX treatment, suggesting that ANPs are part of the pathway targeted by this inhibitor and play a role in cell wall integrity surveillance.

**Highlights**: The loss of ANP function affects cell wall composition and leads to typical cell wall damage-induced phenotypes, such as ectopic lignification and jasmonic acid accumulation.

## Introduction

One of the distinctive features between plants and animals is the presence of chemically complex rigid cell walls. This structure surrounds the cells and provides support as well as protection against biotic and abiotic stresses. The plant cell wall is also a dynamic compartment involved in cell–cell and cell–environment interactions as well as in the control and diffusion of signaling molecules that are perceived at the cell surface (Bashline et al., 2014; Malinovsky et al., 2014). It is mainly a carbohydrate-based complex structure, in which a scaffold of cellulose (a linear β-1,4-glucan) microfibrils is embedded in a matrix made of heterogeneous and branched polysaccharides and a smaller proportion of proteins, organized to form a porous network (Caffall and Mohnen, 2009). Matrix polysaccharides include neutral hemicelluloses, which are tightly bound to cellulose and pectin, an acidic galacturonic acid-containing polymer that represents the more external component of the wall and is bound mostly through Ca^++^ bridges (Caffall and Mohnen, 2009). The wall components are finely remodeled during plant growth and tissue expansion, and differ in their composition, dimension and elasticity between cells and tissue types, depending on tissue function and developmental stages. Cell wall components, like pectin, are produced in the Golgi and together with cell wall-associated proteins, including cellulose synthases (CESAs), callose synthases (CALSs) and pectin methylesterases (PMEs), are transported to the plasma membrane *via* exocytosis (Kim and Brandizzi, 2014). To generate and/or modify cell walls, plants use secretory and endocytic pathways; processes also involved in the generation of the cell plate during cell division (McMichael and Bednarek, 2013; Jürgens et al., 2015) and in the delivery and/or in the internalization of cell wall components.

Plants are able to perceive the modifications to cell wall composition through complex mechanisms devoted not only to the detection but also to the induction of responses to cell wall damage (CWD) (Denness et al., 2011; Engelsdorf and Hamann, 2014). Even if the nature of the CWD is different, its perception activates intracellular signaling cascades leading to responses that are often activated in immunity. In plants, perception of Microbe-Associated Molecular Patterns, (MAMPs) as well as Damage-Associated Molecular Patterns (DAMPs), activates plant responses, collectively termed immune responses (Schwessinger et al., 2011). For example the DAMPs oligogalacturonides (OGs), pectin fragments derived from homogalacturonan (HG), and cellodextrines, cellulose-derived oligomers, are both considered to be perceived as indicators of tissue injury (Brutus et al., 2010; Ferrari et al., 2013; Senechal et al., 2014; Benedetti et al., 2015; Souza et al., 2017).

The Arabidopsis Nucleus- and Phragmoplast-localized protein Kinase 1-related Protein kinases (ANP1, ANP2 and ANP3) (Krysan et al., 2002) have been implicated in the signaling cascade activated by OG- and MAMPs-triggered immunity (Savatin et al., 2014). We have previously shown that these three kinases are required for the OG-induced phosphorylation of the mitogen-activated protein kinases MPK3 and MPK6, reactive oxygen species (ROS) accumulation and induction of defense gene expression (Savatin et al., 2014). ANPs have been initially described to be essential for Arabidopsis growth, since *anp1 anp2 anp3* triple mutants, generated by crossing, are embryo-lethal (Krysan et al., 2002). These kinases are putative orthologs of the *Nicotiana tabacum* kinase NPK1 (Krysan et al., 2002), a protein required for the progression of cytokinesis in tobacco (Nishihama et al., 2002; Tanaka et al., 2004). However, among the double mutant combinations, only *anp1 anp3* and *anp2 anp3* display developmental defects (Krysan et al., 2002). In *anp1 anp3* the defects are mainly in the floral organs while in *anp2 anp3* growth is severely affected and root length is reduced, accompanied by slight root cell bulging. *anp2 anp3* tissues also show scattered multinucleate cells, due to incomplete cell wall formation (cell wall stubs), implying that ANPs, like NPK1, are also involved in cytokinesis (Krysan et al., 2002). Moreover, the phenotype of the *anp2 anp3* double mutants is similar to that of mutants lacking MPK4 (*mpk4*), which is also required for the regulation of immunity. It has been shown that MPK4, which, like NPK1, localizes to the phragmoplast, physically interacts with the Microtubule Associated Protein 65 (MAP65-1), a microtubule-crosslinking protein that regulates the bundling, stability and the turnover of phragmoplast microtubules (Krysan et al., 2002; Beck et al., 2010, 2011). Recently, the slight root epidermal cell bulging observed in the double *anp2 anp3* mutants, has been linked to microtubule bundling due to excessive microtubule stabilization (Beck et al., 2010). It has also been proposed that ANPs interact with proteins such as the kinesin-like protein HINKEL (HIK) that has been proposed to activate MAP65-1, involved in the regulation of microtubule stability and turnover during cytokinesis (Takahashi et al., 2010; Komis et al., 2011). Whether ANPs localize to the phragmoplast is not yet known.

In this paper we addressed the basis of the root alterations observed in the *anp* mutants (described in Krysan et al., 2002) using conditional *anp* loss-of-function mutants previously generated to overcome the problem of lethality in triple knock-out (KO) mutants (Savatin et al., 2014). The conditional triple *anp* mutants exhibit severe defects in rosette development (Savatin et al., 2014), and we show here that the dramatic seedling root swelling is accompanied by cell wall composition alterations and the so-called cell wall damage syndrome (CWDS), which has been described in plants defective in cell wall composition or cellulose biosynthesis due to pharmacological or genetic inhibition. The cellulose biosynthesis inhibitor isoxaben (ISX) has been studied in detail to dissect the response to cell wall damage, which includes the production of ROS, phytohormone accumulation and, later, lignin deposition (Hamann et al., 2009; Denness et al., 2011). Based on our observations, the triple *anp* mutant phenotype mimics the ISX-induced phenotype in many details, pointing to a role for these kinases in the CW integrity maintenance mechanism.

## Materials and Methods

### Plant Growth and Treatment

Arabidopsis (*Arabidopsis thaliana*) Wassilewskija (Ws) wild type seeds were purchased from Lehle Seeds. The *anp* double mutants (in the Ws background) mutants were kindly provided by Patrick J. Krysan (University of Wisconsin). Generation of conditional triple mutants [amiR1 (line 2.5), and amiR3 (line 5.3)] and of plants overexpressing *ANP1* (OE1, line 2.15) or *ANP3* (OE3, line 3.1) has been reported previously (Savatin et al., 2014). For gene expression analysis, seeds were surface sterilized and germinated in multiwell plates containing 0.5X Murashige and Skoog medium (1 ml/well; Sigma–Aldrich) supplemented with 0.5% (w/v) sucrose. Triple mutant seedlings were grown in 0.5X MS containing β-estradiol (1 μM for wild type and amiR1, 10 nM for amiR3) or DMSO (mock; 0.01% v/v) for 9 days. The culture medium was then replaced with fresh medium containing β-estradiol at the same concentrations and seedlings were grown for further 24 h before the analysis. For phenotypical analysis of the triple mutants roots, 5-day-old seedlings of wild type, amiR1 and amiR3 grown in presence/absence of estradiol at different concentrations (0.5–1 μM for amiR1, 2.5–50 nM for amiR3) in agar plates containing 0.5X Murashige and Skoog medium supplemented with 0.5% (w/v) sucrose. In the case of ISX treatment for Jasmonate analysis, 6 day-old seedlings of Ws, *anp1 anp2*, *anp1 anp3*, *anp2 anp3* or Col-0, OE*ANP1* and OE*ANP3* were grown in liquid 0.5X Murashige and Skoog medium supplemented with 1% (w/v) sucrose. Cell wall analysis was performed in roots collected from 10-day-old seedlings of wild type, amiR1 and amiR3 grown in 0.5X MS plates in the presence of 1 μM estradiol or DMSO, and wild type seedlings treated for 24 h with 20 nM ISX (*N*-[3-(1-ethyl-1-methylpropyl)-1,2-oxazol-5-yl]-2,6-dimethoxybenzamide-PESTANAL^®^, Sigma–Aldrich). For the latter treatment, wild type seedlings were grown for 9 days in plates containing DMSO and then transferred for 24 h to medium containing 20 nM ISX.

### Root Cell Wall Analysis

Roots were collected, washed extensively with distilled water to remove sugars contained in the medium, weighed, frozen and milled using a Retschmill MM300 for 60 s at 18 Hz. The root powder was suspended in 1 ml of pre-warmed (70°C) 70% ethanol and left for 30 min at room temperature (25°C). Samples were centrifuged at 25000 × *g* for 10 min (Beckman Allegra) to pellet the Alcohol Insoluble Solid (AIS). The supernatant containing pigments were discarded. In order to eliminate proteins and nucleic acids the AIS was suspended in 1 ml chloroform:methanol (1:1) and incubated again for 30 min at room temperature. Samples were centrifuged at 25000 × *g* for 10 min (Beckman Allegra) and supernatant was discarded. This step was repeated twice. To remove lipids and facilitate subsequent dehydration the pellet was washed in 1 ml 80% acetone, recovered by centrifugation at 25000 × *g* for 5 min. This step was repeated twice. The pellet was dried O/N at room temperature under a fume hood.

### Starch Removal

The AIS material obtained as described above was weighed and transferred in 2-ml screw-capped Eppendorf tubes. Buffer 1 (100 mM phosphate buffer, 5 mM NaCl, sodium azide 0.02% w/v, pH 7.0; 500 μl) was added and samples were heated at 70°C for 10 min to gelatinize starch granules. The suspension was cooled at 4°C for 10 min and 1.2 μl of α-amylase Type I-A (Sigma–Aldrich; 5U/mg AIS) was added to each sample. To improve digestion, samples were incubated in rotary shaker at 37°C for 24 h as previously described (Zablackis et al., 1995). After 24 h, samples were centrifuged at 25000 × *g* for 10 min and supernatants were discarded. The de-starched AIS (ds-AIS) was washed twice with 1 ml of water, centrifuged at 25000 × *g* and the pellet was washed once with 80% acetone. Samples were centrifuged at 25000 × *g* for 5 min and supernatant was eliminated. Pellet was dried O/N at room temperature under a fume hood.

### Quantification of Total Sugars

Root cell wall material was subjected to total hydrolysis (according to Saeman et al., 1954). Briefly, the de-starched AIS was weighed and treated firstly with 175 μl of 72% H_2_SO_4_ at 30°C for 90 min. The sample was diluted to a sulphuric acid concentration of 13% by adding 825 μl of H_2_O in screw capped tubes and incubated at 121°C for 90 min. For monosaccharide composition, samples were neutralized with 0.1 M Ba(OH)_2_, centrifuged twice at 8000 × *g* for 10 min to remove BaSO_4_. Supernatants were filtered and analyzed directly by HPAEC-PAD. Sugar content of the ChASS, 0.1K-H/P and 4K-H fractions was determined according to (Dubois et al., 1956).

### Extraction and Precipitation of Pectin-Enriched Fraction ChASS (Chelating Agent-Soluble Solids) and Hemicellulose

The de-starched AIS was incubated with the Chelating Agent Solution (CAS) [50 mM 1,2-diaminocyclohexanetetraacetic acid (CDTA), 50 mM ammonium oxalate, in 50 mM ammonium formiate, (pH 5.2)] for 2 h at 70°C. To improve the extraction, samples were vortexed every 20 min. After 2 h, samples were centrifuged at 14000 × *g* for 5 min. These steps were repeated twice. The supernatants obtained from these subsequent extractions were combined to obtain the ChASS fraction. To remove the CAS from the ChASS fraction, samples were precipitated with EtOH at final concentration of 30% (v/v) under continuous shaking O/N at 4°C. Samples were centrifuged at 25000 × g for 20 min and pellet was dissolved the in 100 μl of ultrapure water. Aliquots of 10 and 20 μl were used to quantify the amount of sugars contained in the ChASS fraction (Dubois et al., 1956).

The residual pellet that contains cellulose, hemicellulose and lignin was treated sub-sequentially with alkali solution (0.1N KOH and 4N KOH) to solubilize hemicellulose contained in the cell wall material as previously described (Peng et al., 2000). After the extraction the pectin/hemicellulose [0.1K-H/P (0.1N KOH)] and the hemicellulose [4K-H (4N KOH)] enriched fraction were dialyzed (vs. water), lyophilized, suspended in water and filtered. Ten and Twenty microliter were used to quantify the amount of sugars contained in these fractions as previously described (Dubois et al., 1956). The ChASS, 0.1K-H/P and 4K-H fractions were firstly hydrolyzed for 16 h with 3N methanolic HCl (Sigma–Aldrich CAS Number 7647-01-0) at 80°C and then with 2 M TFA. After evaporation of the TFA under a stream of N_2_ by adding 2-propanol, samples were dissolved in distilled water and used for HPAEC-PAD analysis.

### Crystalline Cellulose Determination

Crystalline cellulose was determined as previously described (Updegraff, 1969). The AIS prepared as described above, was treated firstly with 2 M TFA, then with Updegraff reagent (acetic acid: nitric acid: water, 8:1:2 v/v) at 100°C for 30 min. Samples were cooled on ice for 20 min and then centrifuged at 14000 × *g* for 10 min. The pellet was washed twice with water and once with 1 ml 80% acetone, and allowed to dry completely on the bench O/N. Dry pellets were subjected to a complete Saeman hydrolysis (Saeman et al., 1954). Glucose in the hydrolysate was analyzed by using HPAEC-PAD after H_2_SO_4_ neutralization with Ba(OH)_2_ as described above.

### ROS Quantification

Basal accumulation of H_2_O_2_ was analyzed in 10-day-old seedlings grown in liquid medium in the presence of 1 μM β-estradiol or DMSO, by using a colorimetric assay based on xylenol-orange as previously described (Galletti et al., 2008).

### Hormone Determination

Jasmonate (JA) was extracted as previously described (Savatin et al., 2014) from 10-day-old seedlings of wild type, amiR1 and amiR3 grown in multiwells containing liquid medium in the presence of 1 μM β-estradiol (wild type and amiR1) or 10 nM β-estradiol (amiR3) or DMSO as a control and quantified using a calibration curve. Statistical differences resulted from the mean value of JA level of three different biological replicates each composed by 40 seedlings (*n* = 40).

ISX-induced JA was analyzed as previously described (Forcat et al., 2008). Seedlings of *anp* double mutants and OE1 or OE3 lines were treated with 20 nM ISX for 7 h, flash-frozen in liquid nitrogen and freeze-dried for 24 h. Extraction buffer (10% methanol, 1% acetic acid) containing internal standards (10 ng jasmonic-d5 acid; CDN Isotopes, Pointe-Claire, QC, Canada) was used for JA extraction. Supernatants obtained from two sequential extractions were combined and centrifuged to remove all debris prior to LC-MS/MS analysis. Chromatographic separation was carried out on a Shimadzu UFLC XR, equipped with a Waters Cortecs C18 column (2.7 mm, 2.1 × 100 mm). The solvent gradient (acetonitrile (ACN)/water with 0.1% formic acid each) was adapted to a total run time of 7 min: 0–4 min 20% to 95% ACN, 4–5 min 95% ACN, 5–7 min 95% to 20% ACN; flow rate 0.4 ml/min.

Ethylene produced by 10-day-old seedlings of wild type, amiR1 and amiR3 grown in 10-ml flasks containing 2 ml of 0.5X MS containing DMSO or β-estradiol (1 μM for wild type and amiR1, 10 nM for amiR3) was quantified as previously described (Brutus et al., 2010).

### Gene Expression Analysis

Gene expression analysis was performed on 10-day-old seedlings of Ws, amiR1 and amiR3 grown in the presence/absence of 1 μM β-estradiol, as previously described in Savatin et al. (2014).

### Lignin Detection

Lignin detection was performed on 5-day-old seedling roots [Ws and amiR3, grown in the presence of β-estradiol (10 nM) or in its absence (DMSO)] cleared overnight in 70% EtOH. A saturated solution of phloroglucinol (in 20% HCl) (Liljegren et al., 2002) was applied on microscope slides, roots incubated for 2 min and covered with coverslips before microscope imaging.

### Cell Wall Stub Analysis

Five-day-old seedlings of Ws, amiR1 and amiR 3 were grown on 0.5X MS in the presence/absence of β-estradiol (1 μM for Ws and amiR1, 50 nM for amiR3). Cotyledons were stained with propidium iodide (P4170- Sigma Aldrich) at a final concentration of 1 mg/ml for 2 min, washed twice with Milli-Q water and imaged with Leica SP8 confocal microscope, excited with a 514-nm laser/610-627 emission. Z-stacks were transformed into Z-projections and cell outlines analyzed by using Fiji (ImageJ).

### Cell Wall Staining

Aniline blue stain was used to visualize callose dots in 10-day-old seedling roots of Ws, amiR1 and amiR3 grown and germinated on 0.5xMS Agar plates containing β-estradiol (1 μM). For callose staining, roots were incubated in 0.07 M sodium phosphate buffer pH 9 for 30 min and in 0.005% (w/v) aniline blue (in 0.07 M sodium phosphate buffer pH 9) for 1 h. After two washes with water, samples were analyzed under a Nikon Eclipse E800 microscope using a UV-2A filter (EX 330–380 nm, DM 400 nm, BA 420 nm). Roots were stained with calcofluor white (in 10% KOH) (18909 Sigma–Aldrich) for 2 min before imaging. For radial sections, roots have been embedded in 3% low melting agarose after calcofluor white staining and hand cut with a razor blade. Images were taken with LSCM Zeiss 800 [405 nm (EX)/450–465 nm (EM)]. Pectin staining was performed on radial sections by using the HG-specific probe COS^488^ (Mravec et al., 2014). Buffer (50 mM MES, pH 5.7) containing COS^488^ was applied on top of the sections for 15 min after which they were washed with 50 mM MES buffer (pH 5.7) prior to visualization. Images were taken with LSCM Zeiss 800, was excited with a 488-nm laser line and emission analyzed between 517 and 527 nm.

## Results

### Roots of Arabidopsis Seedlings Lacking ANP Function Display Root Elongation Impairment Likely Due to Cell Bulging in the Epidermis of the Transition Zone

**FIGURE 1:**
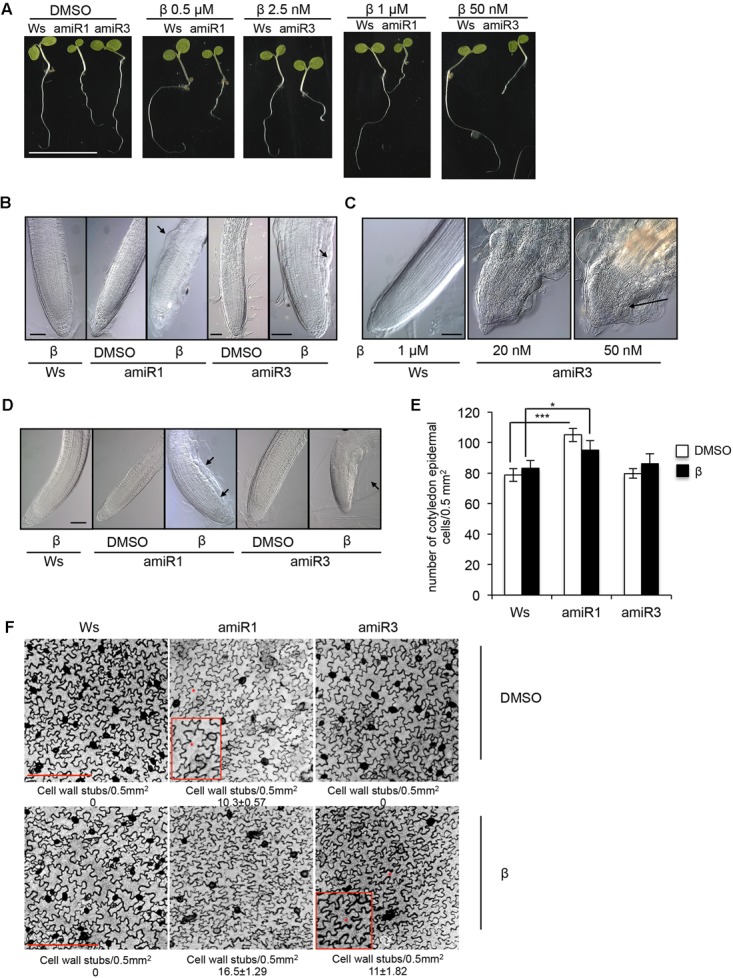
Phenotypes of the triple *anp* mutants. **(A)** Phenotypic characterization of 5-day-old seedlings of triple mutants amiR1 and amiR3 germinated and grown in the presence of β-estradiol display root growth inhibition. Bar = 1 cm. **(B)** Five-day-old seedlings of triple mutants amiR1 and amiR3 grown in the presence of β-estradiol (β; 0.5 μM and 2.5 nM respectively) display epidermal cell bulging (black arrows) in the transition zone of the primary root. Bar = 100 μm. **(C)** Five-day-old seedling roots of the triple amiR3 mutants grown in the presence of 20 or 50 nM β-estradiol display severe alterations in the organization of primary root tip tissues. Bar = 100 μm. **(D)** Lateral root phenotype of 15-day-old seedlings germinated and grown in the presence or in the absence of β-estradiol at the same concentrations as in **(A)**. Bar = 100 μm. **(E)** Epidermal cell number analyzed in 5-day old seedling cotyledons of wild type and amiR1 grown in the presence/absence of 1 μM β-estradiol, amiR3 (50 nM β-estradiol). Cell number has been analyzed in 5-day-old seedling cotyledons stained with propidium iodide (1 mg/ml) and imaged with LSCM. *n* = 4. Area 0.5 mm^2^. ^∗^*P* < 0.05, ^∗∗∗^*P* < 0.001 according to Student’s *t*-test. **(F)** Representative pictures of epidermal cells showing wall stubs. Cell wall breaks number and related SD, have been measured in seedlings grown and treated as in **(E)**, and expressed as number of cell wall breaks/0.5 mm^2^. Asterisks indicate cell wall stubs and are highlighted in the insets. *n* = 5. Bar = 250 μm. Control wild type seedlings were germinated and grown in the presence of 0.5 μM **(B,C)**, and 1 μM β-estradiol **(D)**.

In this study we used previously described conditional *anp* triple mutants, in which the expression of the third gene family member was silenced in both the a*np1 anp2* and *anp2 anp3* double mutants, by individually expressing *ANP3*- and *ANP1*-specific artificial microRNAs (amiRNAs), respectively, under the control of a β-estradiol-inducible promoter (Savatin et al., 2014). The transgenic lines of each background, homozygous for a single insertion, when induced by β-estradiol, are hereon indicated as triple mutants amiR1 and amiR3 (line 2.5 and line 5.3, respectively). The two types of mutants respond to different concentrations of the inducer (**Figure 1A**), which may be due to different permeability of β-estradiol in the different mutant backgrounds. The overall phenotype of 5-day-old triple *anp* mutant seedlings, germinated and grown in β-estradiol (0.5/1 μM for amiR1 and 2.5/50 nM for amiR3) is shown in **Figure 1A**. While amiR1 control seedlings (labeled as DMSO, β-estradiol solvent) displayed a global development defect (as previously described in Krysan et al., 2002; Savatin et al., 2014), both triple *anp* mutants displayed reduction in primary root elongation when grown in the presence of β-estradiol compared to their corresponding controls (**Figure 1A**). To investigate closely, we analyzed the primary root phenotype in seedlings germinated and grown for 5 days in the presence of intermediate doses of the β-estradiol (0.5 μM for amiR1 and 2.5 nM for amiR3). Primary roots of both amiR1 and amiR3 mutant seedlings showed cell bulging in the epidermis of the transition zone (TZ) (**Figure 1B**, black arrows), suggesting that root length and consequently elongation might be linked to these defects. Nonetheless no alteration was detected in organization of root tip tissues compared to the wild type by using these doses of β-estradiol (**Figure 1B**). However, at higher doses, defects in the root phenotype were more severe and development of the entire root tip was completely compromised in both amiR lines (**Figure 1C**, black arrow). In the latter situation, radial swelling and tissue disorganization have been observed not only in the TZ but also in the meristem; moreover, vascular differentiation was observed in close proximity to the stem cell niche (**Figure 1C**). A similar phenotype was observed in triple mutant lateral roots, where, after 15 days of growth in β-estradiol, cell bulging was visible in the epidermis approximately in the same area as previously shown for triple *anp* mutant primary roots (**Figure 1D**).

We also investigated the impact of the loss of ANPs on cotyledon development, by analyzing epidermal cells in 5-day-old seedling cotyledons of Ws, amiR1 and amiR3 germinated and grown in the presence or absence of 1 μM (Ws and amiR1), or 50 nM β-estradiol (amiR3). We found that, although the lack of these proteins did not affect the number of epidermal cells (**Figure 1E**) thus did not significantly influence the cotyledon size, it altered the number of cell wall stubs (**Figure 1F**). It has been previously reported that the simultaneous loss of *ANP2* and *ANP3*, but not of *ANP1* and *ANP2*, strongly disturbs the formation of complete cell walls, leaving remnant cell wall stubs and multinucleated cells (Krysan et al., 2002). As expected, we observed cell wall stubs in the control DMSO-grown amiR1 (*anp2 anp3*) seedlings, but not in the amiR3 (*anp1 anp2*) ones (**Figure 1F**, upper panel). In the amiR1 seedlings, β-estradiol-induced *ANP1* silencing enhanced the formation of cell wall stubs, which became detectable also in the β-estradiol-induced amiR3 seedlings (**Figure 1F**, lower panel).

### Triple Mutant Roots Display Defects in Cell Wall Polysaccharide Composition and Content

Because the cell bulging phenotype of the triple mutant strongly suggests alterations in the cell walls, we investigated cell wall composition in roots. In the analysis we included wild type roots treated with isoxaben (ISX), because mild treatment with this cellulose biosynthesis inhibitor induces a strikingly similar phenotype (Supplementary Figure S1A). We also observed that 48 h of treatment with β-estradiol was sufficient to trigger the phenotypical defects of the triple *anp* mutants (Supplementary Figure S1B).

**FIGURE 2:**
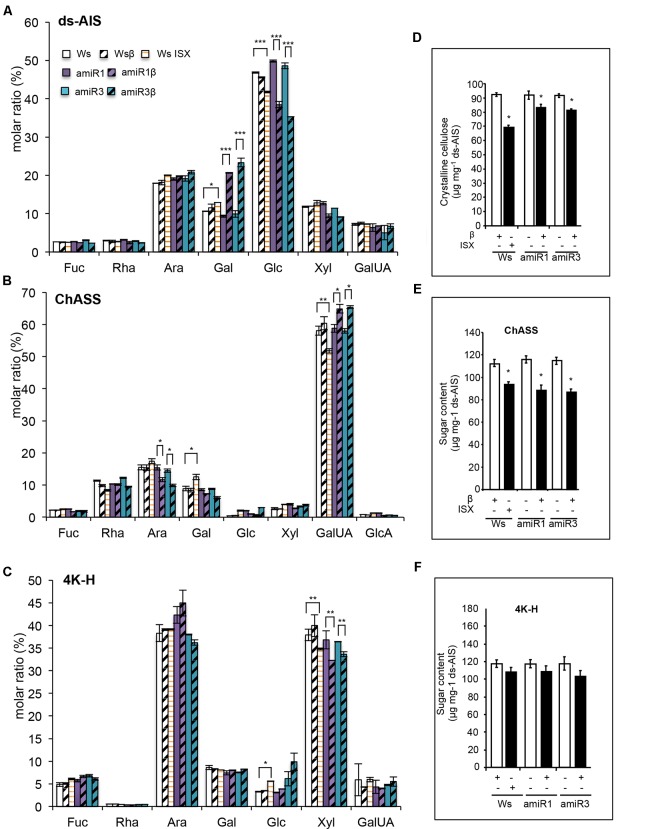
Composition of root cell walls is altered in *anp* triple mutants. **(A)** Monosaccharide composition of the destarched alcohol-insoluble solids (ds-AIS), the pectic fraction (ChASS) **(B)** and the hemicellulose fraction (4K-H) **(C)** extracted from roots of wild type (Ws) and *anp* triple mutants seedlings grown in DMSO (control) or β-estradiol and from ISX-treated wild type seedlings. Molar ratio (%) of each sugar is shown. Bars represent means ± SE (*n* = 4); for each monosaccharide, asterisks indicate statistically significant differences between ISX treated Ws and its control DMSO, β-estradiol grown amiR1 or amiR3 and their corresponding controls (DMSO-grown amiR1 or R3), according to Student’s *t*-test (^∗^*P* < 0.05; ^∗∗^*P* < 0.01, ^∗∗∗^*P* < 0.01). **(D)** Crystalline cellulose content in the ds-AIS. Asterisks indicate statistically significant differences between triple mutants or ISX-treated seedling roots and β-estradiol treated Ws, according to Student’s *t*-test or β-estradiol grown amiR1 or amiR3 and their corresponding controls (DMSO-grown amiR1 or R3) (^∗∗^*P* < 0.001; ^∗^*P* < 0.01). **(E,F)** Abundance of ChASS and 4K-H fractions expressed as sugar content. Asterisks indicate statistically significant as in B according to Student’s *t*-test (^∗^*P* < 0.01). Statistical differences have been evaluated comparing ISX-treated Ws and its corresponding control and β-estradiol grown amiRs and their controls (amiR1 β-estradiol vs. amiR1 DMSO, or amiR3 β-estradiol vs. amiR3 DMSO).

To perform the analysis a fraction corresponding to the de-proteinized and de-starched total cell walls (de-starched Alcohol-Insoluble Solid, ds-AIS) was prepared from whole roots of triple mutants and wild type seedlings grown in the presence of β-estradiol (1 μM) or DMSO (mock), as well as of wild type seedlings grown for 24 h in the presence of 20 nM ISX. Part of the ds-AIS was subjected to complete hydrolysis (Saeman et al., 1954) for monosaccharide composition (MSC) analysis. Both the triple mutants and ISX-treated wild type roots displayed a significant reduction in the relative amount of glucose [expressed as molar ratio (%)] compared to the corresponding untreated plants (**Figure 2A**). β-estradiol-treated *anp* triple mutant roots were also characterized by an increase of galactose (Gal) (**Figure 2A**). To obtain the different cell wall components, the remaining part of the ds-AIS was subjected to sequential extractions (Peng et al., 2000; Bischoff et al., 2009; Socha et al., 2014). After extracting the fraction mainly composed of pectins (named Chelating-Agent Soluble Solid or ChASS) (**Figure 2B**), a fraction containing both loosely bound hemicellulose and strongly bound pectins (solubilized by using KOH 0.1 M, and named 0.1K-Hemicellulose/Pectin (or 0.1K-H/P)) was extracted (Supplementary Figure S2). Finally, a fraction containing mainly hemicellulose was obtained by solubilization with 4 M KOH (named 4K-Hemicellulose or 4K-H) (**Figure 2C**). In the triple mutants, ChASS showed a significantly decreased relative amount of arabinose (Ara) and increased amount of galacturonic acid (GalUA); after ISX treatment, the relative content of GalUA was instead reduced (**Figure 2B**). The 0.1K-H/P fractions of β-estradiol treated triple mutant roots did not show any significant differences from the control (DMSO treated triple mutants) (Supplementary Figure S2A) whereas the 4K-H fractions of both the triple mutants and the ISX-treated wild type roots exhibited a significant reduction in relative amount of Xyl compared to the corresponding control seedlings (**Figure 2C**). No significant difference in glucose was detected in these two fractions between the triple mutants and the wild type. We then assessed whether the relative lower content of glucose observed in the ds-AIS MSC reflects a decrease content of crystalline cellulose. Indeed, the content of crystalline cellulose was reduced both in ISX-treated wild type roots and in the induced triple mutants when compared with their respective DMSO-treated controls (**Figure 2D**). The total abundance of pectins and loosely bound hemicelluloses/strongly bound pectins (ChASS, 0.1K-H/P) or hemicelluloses (4K H) was also examined. Both the triple mutant and the ISX-treated wild type root cell walls showed a significant reduction in the abundance of pectins contained in the ChASS fraction (**Figure 2E**). ISX-treated seedlings and induced amiR3 seedlings also showed an increased abundance of the 0.1K-H/P fraction, while the amiR1 mutant had an already increased abundance in the control treatment and no further increase after induction with β-estradiol (Supplementary Figure S2B). No significant decrease in the amount of hemicelluloses contained in the 4K-H fraction was observed in the triple mutants and in the ISX-treated wild type roots (**Figure 2F**).

In order to study the localization of the cell wall defects of the triple *anp* mutants, we analyzed the distribution of callose and cellulose by using aniline blue and calcofluor white, respectively (Supplementary Figures S3A,B). Aniline blue appeared to penetrate into the β-estradiol-grown triple *anp* mutants more easily than into the corresponding DMSO-treated controls. However, the presence of small bright dots (likely representing callose deposits) was observed only upon silencing of *ANP1* or *ANP3* (Supplementary Figure S3A, white arrows). Apart from the bulging phenotype typical of the *anp* mutants, calcofluor white staining did not reveal significant differences between the mutant and the wild type roots in terms of cellulose abundance or patterning (Supplementary Figure S3B). Indeed, the fluorescence intensity of the different stained roots was similar in all the genotypes (Ws, amiR1 and amiR3) and conditions analyzed (DMSO, β-estradiol). We also tested the HG-specific probe COS^488^ on the same seedling roots, to detect the cellular location of HG with different esterification states (Mravec et al., 2014). Interestingly, we found, in the triple *anp* mutant root sections, a reduction of demethylesterified HG, which normally localizes mostly at the corners of intercellular spaces in proximity of cells connection (Supplementary Figure S4, middle panels white arrows), compared to the DMSO-treated amiR controls.

Taken together, these results show that the composition of root cell walls in the β-estradiol-treated triple mutants is severely altered, with an altered pectin composition and a lower content of glucose, as detected in the total cell wall preparations. The latter defect may be ascribed to a reduction in crystalline cellulose content, although it was not revealed by calcofluor white, a stain that, however, binds β-glucans besides cellulose (Pradhan and Loqué, 2014) and, in any case, does not detect subtle changes in cellulose content. It is also possible that an altered organization of cellulose, rather than a decrease abundance, may account for the dramatic phenotype of the triple *anp* mutants.

### Lack of ANP Function Triggers a Typical Cell Wall Damage Syndrome

**FIGURE 3:**
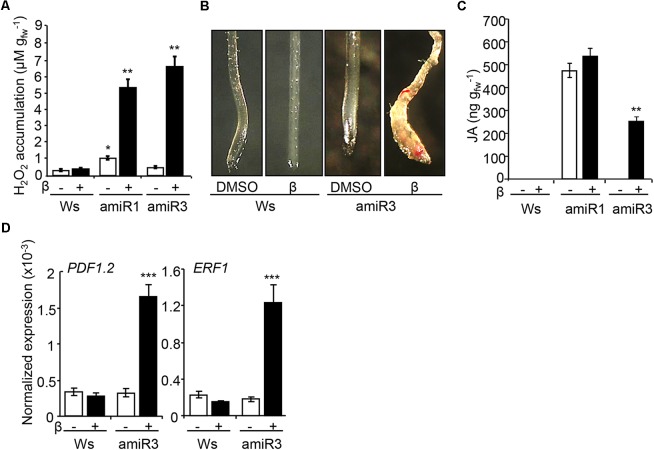
β-estradiol- treated *anp* triple mutants display the cell wall damage syndrome (CWDS). **(A)** ROS accumulation, analyzed in triple mutant seedlings grown in the presence/absence of β-estradiol (1 μM). Data represent the mean values of four independent replicates (±SE). According to Student’s *t*-test, asterisks indicate statistically significant differences between β-estradiol-grown *anp* triple mutant and wild type (Ws) seedlings (^∗∗^*P* < 0.001). DMSO-grown amiR1 seedlings produce *per se* an increased amount of H_2_O_2_ compared to the control seedlings (^∗^*P* < 0.05). *n* = 3. **(B)** Ectopic lignin deposition, analyzed in 5-day old Ws and amiR3 seedlings grown in the presence/absence of β-estradiol (10 nM). Similar results were obtained in the β-estradiol-treated amiR1seedling roots. **(C)** JA levels were measured in 10-day-old Ws and triple mutant seedlings grown in the presence/absence of β-estradiol (1 μM for amiR1, 10 nM for amiR3). Data represent mean values of four independent experiments (±SE). Asterisks according to Student’s *t*-test indicate significant differences between DMSO and β-estradiol (^∗∗∗^*P* < 0.001). **(D)** Expression of *PDF1.2*, and *ERF1* in Ws and amiR3 seedlings grown and treated as in C. Analyses were performed by qRT-PCR and transcript levels are shown as the mean of three independent experiments (±SE; *n* = 20 in each experiment) normalized to *PEX4* expression. Asterisks (^∗∗∗^*P* < 0.001) indicate statistically significant differences according to Student’s *t*-test between expression in the β-estradiol-treated amiR3 and DMSO (amiR3 or wild type).

The perception of the cell wall damage induces complex responses, hereon collectively referred as CWDS, as described in several cell wall mutants or upon cellulose biosynthesis inhibition (Caño-Delgado et al., 2000; Ellis et al., 2002; Denness et al., 2011; Tsang et al., 2011). The CWDS includes ROS accumulation, ectopic lignification of tissues and phytohormone [jasmonic acid (JA) and salicylic acid (SA)] accumulation (Denness et al., 2011). Here, we tested whether the cell wall defects occurring in plants that lack ANPs activate the CWDS, by examining extracellular hydrogen peroxide production, lignification and JA levels. Indeed, higher levels of extracellular hydrogen peroxide were produced by amiR1 and amiR3 triple mutant seedlings, germinated and grown in the presence of β-estradiol (**Figure 3A**). Control DMSO-treated amiR3 seedlings showed hydrogen peroxide levels that were comparable to those detected in the wild type, whereas DMSO-treated amiR1 control seedlings, which exhibit morphological defects, showed higher levels (**Figure 3A**), in agreement with previous results (Savatin et al., 2014). Next, the presence of lignin was analyzed in wild type and amiR3 triple mutant seedlings by staining with phloroglucinol-HCl solution. Triple mutant roots displayed a patchy distribution of staining that was undetectable in the non-induced controls (**Figure 3B**). The ectopic lignification occurred in proximity of the root tip. JA quantification showed that JA was undetectable in non-induced amiR3 plants and increased in the presence of β-estradiol (**Figure 3C**). In contrast, JA content was already high in non-induced amiR1 seedlings compared to both the wild type and non-induced amiR3 seedlings, and did not increase further upon β-estradiol treatment (**Figure 3C**).

Consistent with the increased JA levels observed, the expression of several JA-regulated genes, such as *PLANT DEFENSIN 1.2* (*PDF1.2*) and *ETHYLENE RESPONSIVE FACTOR1* (*ERF1*), was increased in the β-estradiol-induced amiR3 mutants, compared to both the non-induced controls and the wild type (**Figure 3D**). The expression of these genes was constitutively high in the *anp2 anp3* double mutant (Supplementary Figure S5), suggesting that lack of both *ANP2* and *ANP3* is sufficient for the induction of the CWDS to some extent.

Expression of both *PDF1.2* and *ERF1* is positively regulated not only by JA, but also by ethylene (Moffat et al., 2012); however, ethylene production in the triple mutant seedlings was comparable to that detected in the β-estradiol-treated wild type seedlings (Supplementary Figure S6), suggesting that only the higher levels of JA lead to increased expression of these genes in the amiR lines. ISX treatment, in which JA induction but not ethylene accumulation occurs, was used as control for the induction of the analyzed genes (Supplementary Figure S7).

### Alteration of ANP Expression Affects the Response to Isoxaben

**FIGURE 4:**
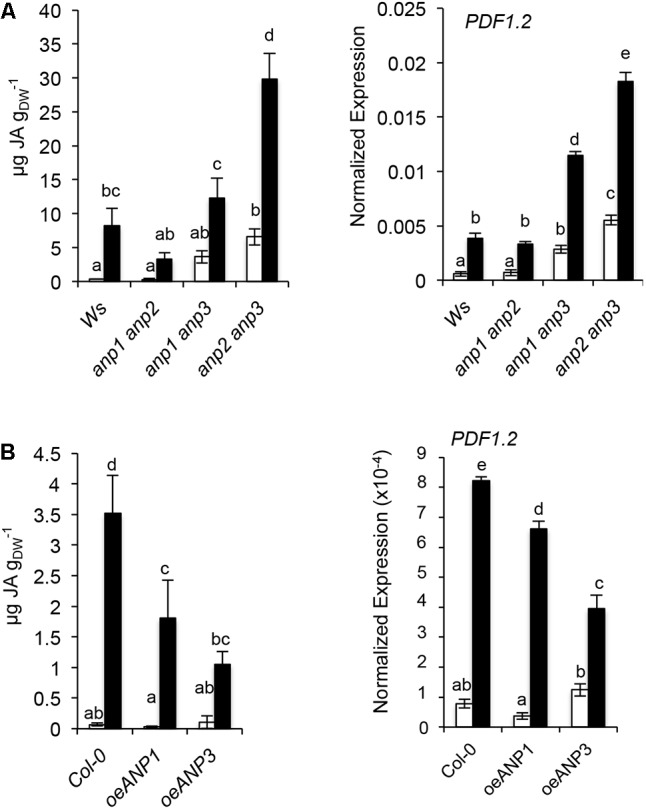
ANP function is required for the regulation of basal and ISX-induced JA level. JA level and *PDF1.2* expression were analyzed **(A)** in wild type (Ws) and *anp* double mutants seedlings or **(B)** in Col-0 seedlings overexpressing ANP1 or ANP3. In both experiments seedlings were grown in liquid cultures containing 0.5X MS and treated with 20 nM ISX (black bars) or DMSO (mock; white bars) for 7 h. JA levels are expressed in μg/g dry weight (DW). Letters represent statistically significant differences according to one-way ANOVA and Tukey’s HSD test (α = 0.05). Values represent the mean of 3 independent biological replicas.

Given the similarity between the ISX-induced phenotypes and the triple *anp* mutants, we investigated the possibility that ANPs play a role in the cell wall integrity (CWI) maintenance pathway. Accumulation of absolute JA levels, a hallmark of the ISX response (Denness et al., 2011), was analyzed in the three combinations of *anp* double mutant seedlings. As shown in **Figure 4A**, basal JA levels and *PDF1.2* expression were wild type-like in *anp1 anp2*, and increased to different degrees in *anp1 anp3* and *anp2 anp3*, which are characterized by developmental defects of differing severity (Krysan et al., 2002). This suggests a positive correlation between the degree of developmental defects and JA accumulation. While *anp1 anp2* showed a response to ISX similar to that of the wild type, *anp1 anp3* seedlings showed an increased expression of *PDF1.2*, and *anp2 anp3* showed both increased JA accumulation and increased *PDF1.2* expression compared to the wild type (**Figure 4A**). We therefore checked whether, conversely, overexpression of *ANP3* or *ANP1* affects the ISX-induced JA levels in the opposite direction. OEANP1 and OEANP3 plants, overexpressing ANP1 and ANP3, respectively, have previously been generated to elucidate the function of these kinases in the responses to DAMPs and MAMPs (Savatin et al., 2014). They displayed wild type-like basal JA levels and *PDF1.2* transcripts, but reduced accumulation of both in response to ISX, with both alterations being more pronounced in OE3 plants (**Figure 4B**). These results indicate that the overexpression of ANP reduces the CWD responses and suggest that these kinases play a role in CWI maintenance.

## Discussion

In the present study, we analyzed the severely defective root phenotype of the conditional β-estradiol-inducible *anp* triple mutants to understand the underlying molecular and cellular alterations and further elucidate the function of ANPs. Loss of these kinases causes severe defects in the TZ along with vascular differentiation in proximity of the root tip; interestingly, unlike the TZ, the root meristem shows defects only at high β-estradiol doses, suggesting that cells of the TZ are more sensitive to the loss of ANP function compared to meristematic cells.

We found that the lack of ANP function triggers typical symptoms of the CWDS, i.e., ectopic lignin deposition, constitutive production of extracellular ROS and accumulation of JA, likely to be secondary effects induced by the alterations in the cell wall. For example, the *anp2 anp3* double mutant used as a background to generate the amiR1 plants, displays *per se* a slight radial root swelling (Beck et al., 2010), an increased level of extracellular ROS and JA (this work) as well as up-regulation of pathogen- and oxidative- related genes (Krysan et al., 2002; Savatin et al., 2014) compared to the wild type, suggesting that in this mutant the CWDS is already triggered. Although less pronounced, the *anp1 anp3* double mutant, previously described to be characterized by mild phenotypic defects, also displays increased basal JA levels compared to the *anp1 anp2*, suggesting that a limited CWDS is induced also in this mutant. It has to be noted that the *anp1 anp2* mutant used as a background for the amiR3 plants displays defects neither at the macroscopic level (root growth/structure) (Krysan et al., 2002) nor at the molecular level (basal intracellular responses such as JA or ROS accumulation) (this work). However, when the silencing of *ANP1* or *ANP3* is induced similar effects take place in both amiR1 or amiR3 plants, i.e., increased ROS levels and lignin deposition in the root, which suggest a strong activation of the CWDS.

Notably, the phenotype of the triple *anp* mutants is similar to that observed after application of ISX (Denness et al., 2011). ISX treatment induces cell bulging and the accumulation of ROS, JA and lignin after 5, 7, and 12 h respectively (Denness et al., 2011). An activated CWDS in *anp* mutants is likely a consequence of cell wall alterations and the perception of the cell wall damage. Indeed, cell wall composition analysis revealed that important defects in the composition of the root cell walls accompany the developmental defects in the triple mutants. A reduced cellulose content and reduced abundance of the pectin (ChASS) fraction, similar to those observed in ISX treated wild type roots, also associated to a higher abundance of the hemicellulose/pectin fraction, characterize the *anp* triple mutant root cell walls. The last feature may be a compensation response (Sugimoto et al., 2003; Lloyd and Chan, 2004; Beck et al., 2010). Not only relative abundance of cell wall polysaccharide components but also their composition is altered in the triple mutants. For example an increased relative amount of galacturonic acid and a reduced arabinose amount characterizes the pectin fraction of the triple mutants; this alteration, however, does not occur in ISX-treated seedlings. This suggests that, besides the similarities with the ISX-induced phenotype (which comprise the reduced crystalline cellulose content), the silencing of the third member of *ANP* gene family induces changes in the cell wall that are more complex than the ones observed in the ISX-treated wild type roots. Here we propose that the vast defects in both cellulose and matrix components shown in *anp* mutant seedlings are the cause of the root cell bulging and swelling phenotype.

The high similarities in phenotype between triple mutants and ISX-treated wild type seedlings suggest that pathway(s) targeted by the herbicide might involve ANPs. This hypothesis is supported by our observations, which show that the ISX-induced accumulation of either JA content or *PDF1.2* transcripts are increased in the *anp1 anp3* and *anp2 anp3* double mutants, and reduced in plants overexpressing *ANP1* or *ANP3*. Our data also shows that the lack of *ANP3* affects to a different extent the basal JA and *PDF1.2* transcript levels depending on the simultaneous loss of either ANP1 or ANP2. This suggests a possible correlation between the degree of phenotypic defects previously observed in these mutants and JA accumulation. Moreover it supports the idea that the members of the family show redundancy to a certain degree, but also have non-redundant functions, both in the generation of macroscopic phenotypes (as already shown in Krysan et al., 2002) and in terms of effect on hormone homeostasis.

It is noteworthy that plant cell growth and shape depend on cell wall composition. Hence, cell wall defects alter cell shape and plant growth. Here we showed that the lack of the MAPKKK family ANPs affects anisotropic growth and crystalline cellulose content. Cellulose, one of the major polysaccharide of the plant primary cell walls, is synthesized at the plasma membrane by the rosette-shaped Cellulose Synthases Complexes (CSCs) (Crowell et al., 2009). Treatment with cellulose biosynthesis inhibitors [ISX or CGA 325′615 (CGA)] has been shown to cause the depletion of CSCs from the plasma membrane (Paredez et al., 2006; Crowell et al., 2009) and the subsequent accumulation of CSCs in small compartments referred to as SmaCCs (Gutierrez et al., 2009) as well as in a subset of the SmaCC population that is associated with microtubules (MASCs; Crowell et al., 2009). It has been demonstrated that CSC movement along cortical microtubules is mediated by the Cellulose Synthase Interacting protein1 (CSI1/POM2) (Li et al., 2012), a scaffold protein required for the anchorage between the CSCs and microtubules. Thus microtubules dynamic instability has been shown to alter CESA behavior (reviewed in Bringmann et al., 2012) compromising microtubule-based trajectories of the CSCs. As previously shown (Beck et al., 2010), the absence of *ANP2* and *ANP3* interferes with microtubule stability. This feature has been suggested to be likely dependent on the ANP-dependent regulation of the functionality of the Microtubule Associated Protein 65 (MAP65-1). The requirement of ANP function for correct microtubule organization might explain their role in the maintenance of cell wall integrity and development as well as in immunity. It would be interesting to determine whether ISX-dependent microtubule defects are dependent or independent from MAP alterations. So far, other MAP kinases have been associated with the regulation of cytoskeleton dynamic/stability (Mazzoni et al., 1993; Zarzov et al., 1996; Rousseau et al., 1997; Delley and Hall, 1999), and the interaction and the possible relation between cytoskeleton and MAPKs has been shown also in plants, despite the limited number of studies. After sensing extracellular stimuli, structural cytoskeletal rearrangements occur through MAPK-mediated phosphorylation of cytoskeleton-associated proteins (Samaj et al., 2004). Like ERK1, the alfa alfa protein kinase SIMK has been found localized to cytokinetic mitotic microtubules [i.e., pre-prophase bands (PPBs) and phragmoplast] (Samaj et al., 2004). Interestingly, in tobacco the ANP homolog NPK1, whose function has been found related to mitotic cell plate formation, localizes to the phragmoplast during cell division. The discovery that the microtubule-associated kinesin-like protein NACK1 regulates the activation and the correct localization of NPK1 on the cell plate (Nishihama et al., 2002) opens the possibility that MAPKKK can interact with elements involved in intracellular transport and not only regulate cytoskeleton stability. MAPKKK are described to act upstream of MAPKK, their direct targets, and MAPKs. Thus, MAPKKKs such as ANPs are likely to represent central hubs, regulating the phosphorylation of MAPKKs and MAPKs involved in many different pathways. Although ANP interactors remain to be identified, a complex scenario emerges where several secondary effects are also likely to play an important role in generating the phenotypes observed. For example, impaired ROS homeostasis in the *anp* mutants (Savatin et al., 2014) might also affect tubulin polymer formation (Livanos et al., 2012; Schmidt et al., 2016).

Collectively, our data suggest that ANPs play an important role in cell wall integrity maintenance and/or protect from cell wall damage. Further studies are necessary to clarify whether ANPs are direct or indirect targets of the ISX-induced cell wall damage or ANPs and ISX treatment share the same downstream targets.

## Author Contributions

NG, DS, and GDL contributed to experimental design and generated data. NG and GDL co-wrote the manuscript. TE contributed with experimental data. FC and GDL supervised this work.

## Conflict of Interest Statement

The authors declare that the research was conducted in the absence of any commercial or financial relationships that could be construed as a potential conflict of interest.
