# Corrigendum: Loss of the Arabidopsis Protein Kinases ANPs Affects Root Cell Wall Composition, and Triggers the Cell Wall Damage Syndrome

**DOI:** 10.3389/fpls.2018.01079

**Published:** 2018-07-18

**Authors:** Nora Gigli Bisceglia, Daniel V. Savatin, Felice Cervone, Timo Engelsdorf, Giulia De Lorenzo

**Affiliations:** ^1^Dipartimento di Biologia e Biotecnologie “C. Darwin”, Istituto Pasteur-Fondazione Cenci Bolognetti, Sapienza Università di Roma, Rome, Italy; ^2^Department of Biology, Norwegian University of Science and Technology (NTNU), Trondheim, Norway

**Keywords:** ANPs, MAP kinase kinase kinase, cell wall damage, root swelling, cellulose deficiency, isoxaben

In the published article, there was an error regarding the affiliation for Daniel V. Savatin. The affiliation “^3^VIB Department of Plant Systems Biology, Ghent University, Ghent, Belgium” should be deleted. The correct affiliation is “^1^Dipartimento di Biologia e Biotecnologie “C. Darwin”, Istituto Pasteur-Fondazione Cenci Bolognetti, Sapienza Università di Roma, Rome, Italy”. “VIB Department of Plant Systems Biology, Ghent University, Ghent, Belgium” is the present address for Daniel V. Savatin. The authors apologize for this error and state that this does not change the scientific conclusions of the article in any way.

The original article has been updated.

## Conflict of interest statement

The authors declare that the research was conducted in the absence of any commercial or financial relationships that could be construed as a potential conflict of interest.

