# Supplementary material for: Loss of the Arabidopsis Protein Kinases ANPs Affects Root Cell Wall Composition, and Triggers the Cell Wall Damage Syndrome
Source: Front Plant Sci. 2018 Jan 22;8:2234. doi: 10.3389/fpls.2017.02234 (PMC5786559; doi:10.3389/fpls.2017.02234)
Supplement: Supplementary file 1 [file Image_1.PDF]

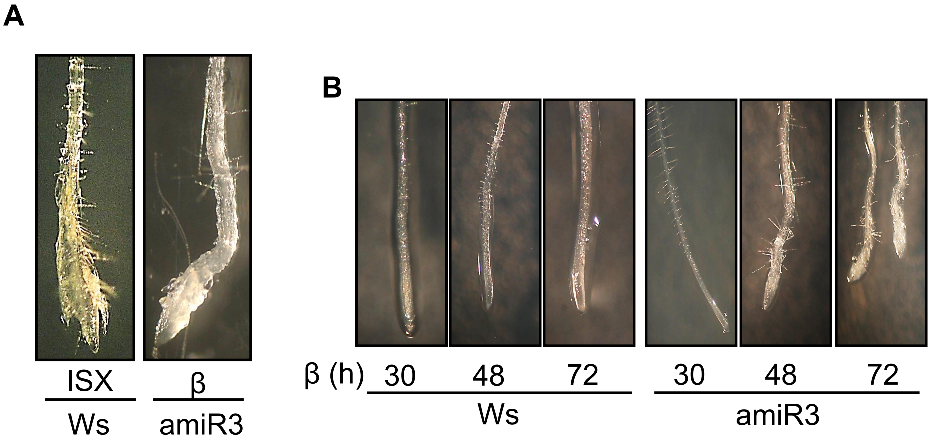

Fig. S1 Similarity between ISX treated wt roots and *anp* triple mutants. (A) Wild-type (Ws) seedlings grown for 6 days in 0.5X MS were transferred for an additional day in 0.5X MS plus 20 nM ISX. AmiR3 seedlings grown for 5 days were transferred in plates containing 1 μM β-estradiol for 72 h. (B) Phenotype of five-day-old seedlings of amiR3 grown in 0.5X MS agar plates and transferred to 0.5X MS agar plates containing 1 μM β-estradiol. Bulging cells and radially expanded roots were clearly visible after 48 h after transfer.
