# Supplementary material for: Loss of the Arabidopsis Protein Kinases ANPs Affects Root Cell Wall Composition, and Triggers the Cell Wall Damage Syndrome
Source: Front Plant Sci. 2018 Jan 22;8:2234. doi: 10.3389/fpls.2017.02234 (PMC5786559; doi:10.3389/fpls.2017.02234)
Supplement: Supplementary file 2 [file Image_2.PDF]

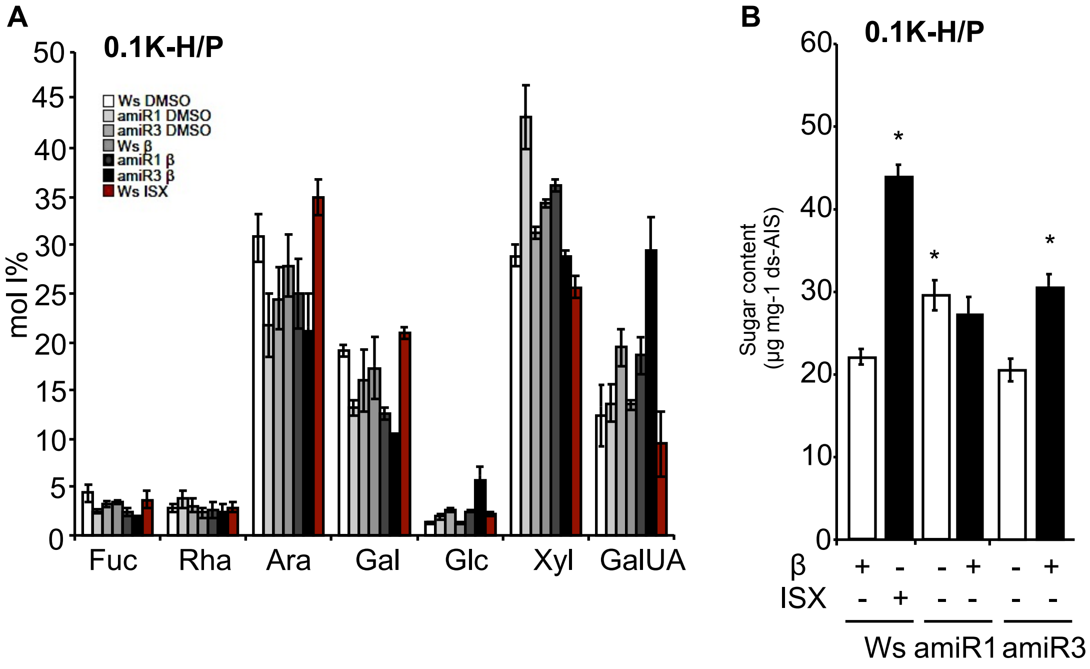

Fig. S2. Monosaccharide composition and sugar abundance of 0.1K-H/P fraction (loosely bound hemicellulose/pectin). (A) Monosaccharide composition of loosely bound hemicellulose/pectin fraction (0.1K-H/P). The relative amount of each sugar is expressed in molar ratio [mol%]. Bars represent means ± SE (n = 4). (B) Abundance of 0.1K-H/P fraction expressed as µg of total sugar per mg of ds-AIS. Asterisks indicate statistically significant as before according to Student's t test (*, $P < 0.01$).
