# Supplementary material for: Loss of the Arabidopsis Protein Kinases ANPs Affects Root Cell Wall Composition, and Triggers the Cell Wall Damage Syndrome
Source: Front Plant Sci. 2018 Jan 22;8:2234. doi: 10.3389/fpls.2017.02234 (PMC5786559; doi:10.3389/fpls.2017.02234)
Supplement: Supplementary file 3 [file Image_3.pdf]

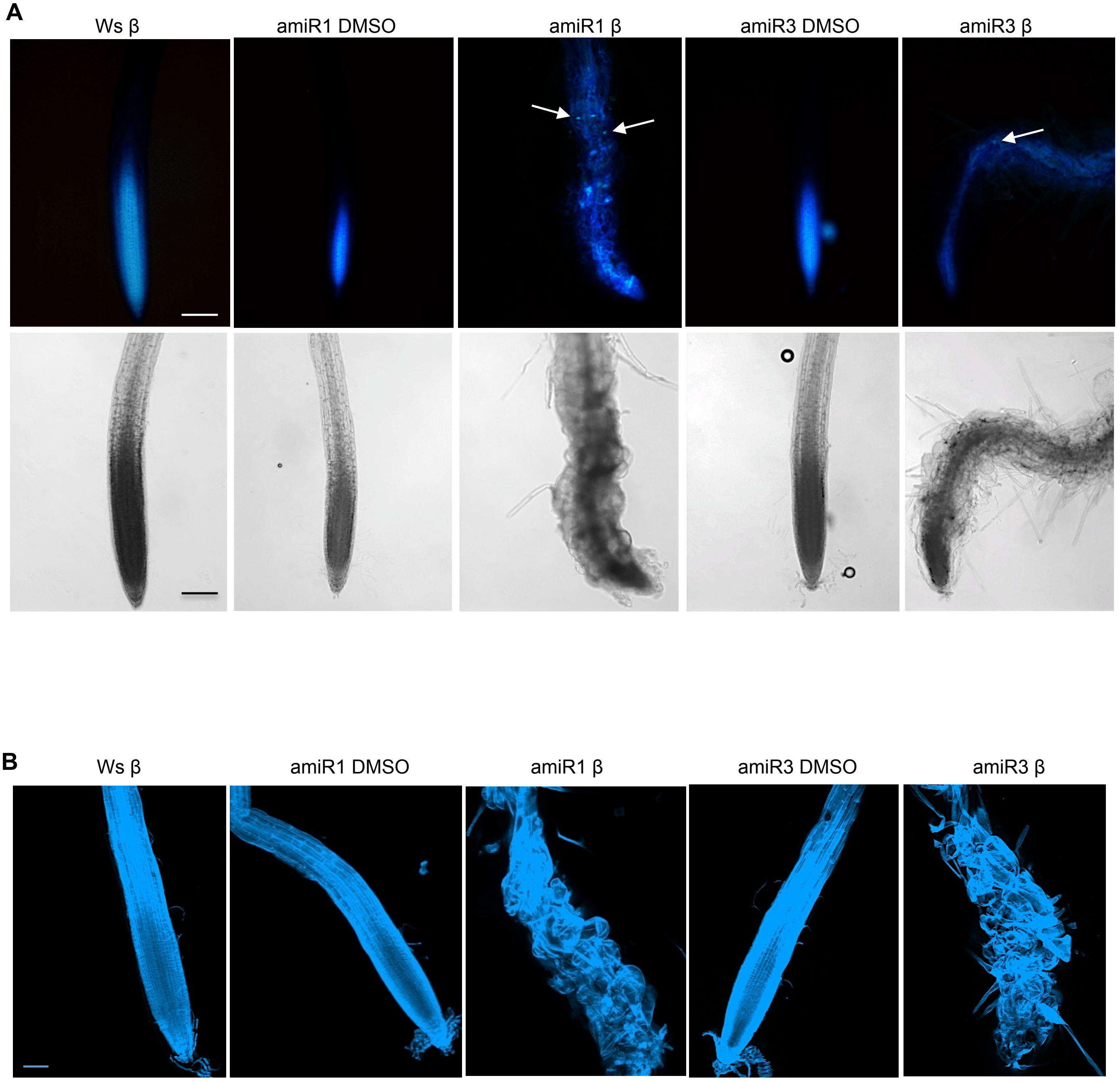

Fig. S3. Callose and cellulose staining in 10-day old *anp* mutant seedling roots. (A) 10-day-old Ws, amiR1 and amiR3 seedlings were grown in the presence or absence of 1 µM β-estradiol and stained for callose detection. White arrows indicate callose dots. Lower panel shows brightfield images. Bar length 150 µm. (B) Calcofluor white was used to stain cellulose in seedlings grown and treated as in A. Bar length 150 µm.
