# Supplementary material for: Loss of the Arabidopsis Protein Kinases ANPs Affects Root Cell Wall Composition, and Triggers the Cell Wall Damage Syndrome
Source: Front Plant Sci. 2018 Jan 22;8:2234. doi: 10.3389/fpls.2017.02234 (PMC5786559; doi:10.3389/fpls.2017.02234)
Supplement: Supplementary file 4 [file Image_4.PDF]

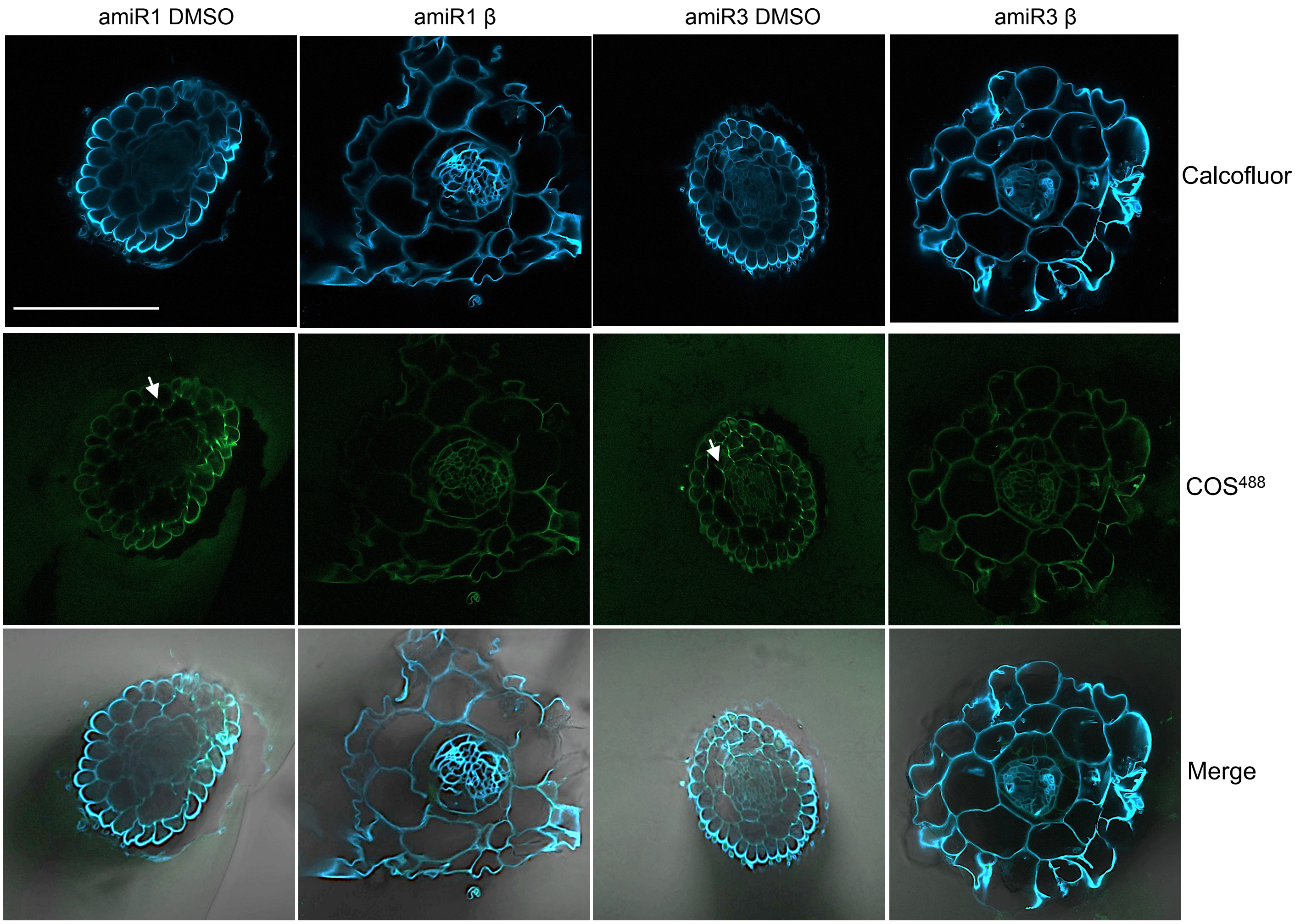

Fig. S4. Pectin staining in 10-day old *anp* mutant seedling roots. Hand cut root sections of amiR1 and 3 grown in the presence or absence of 1 μM β-estradiol were stained with calcofluor white or COS^{488}. White arrows indicate cell connections marked with the demethylesterified HG-specific probe. Bar length 130 μm.
