# Supplementary material for: Loss of the Arabidopsis Protein Kinases ANPs Affects Root Cell Wall Composition, and Triggers the Cell Wall Damage Syndrome
Source: Front Plant Sci. 2018 Jan 22;8:2234. doi: 10.3389/fpls.2017.02234 (PMC5786559; doi:10.3389/fpls.2017.02234)
Supplement: Supplementary file 5 [file Image_5.PDF]

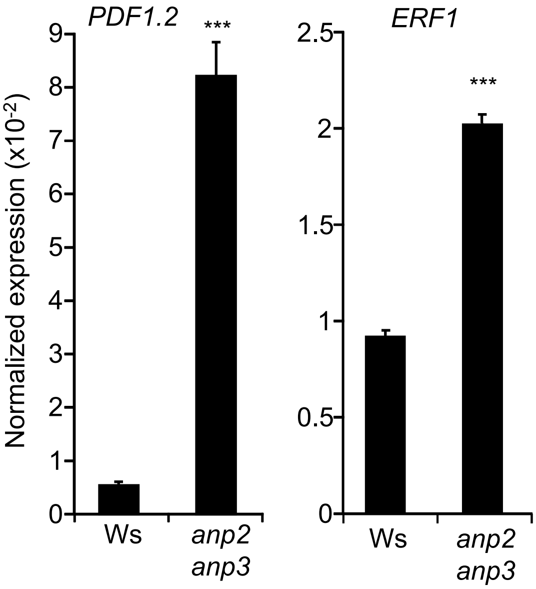

Fig. S5. Basal expression of JA-regulated marker genes in double *anp2 anp3* KO mutant. Basal expression of *PDF1.2* and *ERF1* was analyzed in 10-day-old wild type and *anp2 anp3* seedlings and found to be up-regulated in the mutant. Analyses were performed by qRT-PCR and transcript levels are shown as the mean of three independent experiments (± SE; n = 20 in each experiment) and normalized to *PEX4* expression. Student's T-test was performed, and asterisks indicate the statistical differences between the basal expression of the JA-related marker gene induction in the *anp2 anp3* relative to wild type seedlings (***, $P<0.001$).
