# Supplementary material for: Loss of the Arabidopsis Protein Kinases ANPs Affects Root Cell Wall Composition, and Triggers the Cell Wall Damage Syndrome
Source: Front Plant Sci. 2018 Jan 22;8:2234. doi: 10.3389/fpls.2017.02234 (PMC5786559; doi:10.3389/fpls.2017.02234)
Supplement: Supplementary file 6 [file Image_6.PDF]

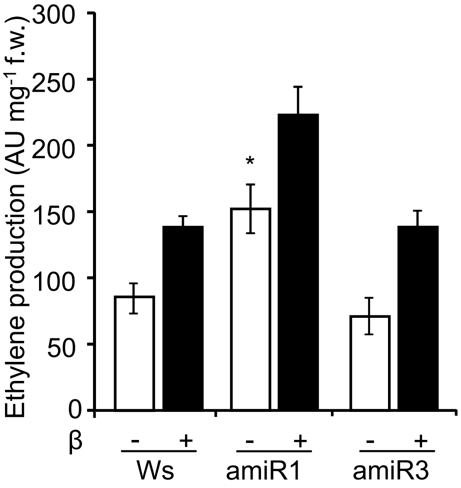

Fig. S6. Ethylene production in *anp* triple mutants. Triple mutants do not produce more ethylene compared to the wild type treated with estradiol. Ethylene production was measured by gas chromatography (GC) in seedlings of wild type and triple mutants grown for 10 days in 10 ml vials containing 0.5X MS in the presence/absence of β-estradiol (1 μM for amiR1 and wild type, 10 nM for amiR3). Data are the means of four replicate samples (±SE). Asterisk indicates statistically significant differences between DMSO treated amiR1 and DMSO treated wild-type seedlings (*, $P<0.001$).
