# Supplementary material for: Loss of the Arabidopsis Protein Kinases ANPs Affects Root Cell Wall Composition, and Triggers the Cell Wall Damage Syndrome
Source: Front Plant Sci. 2018 Jan 22;8:2234. doi: 10.3389/fpls.2017.02234 (PMC5786559; doi:10.3389/fpls.2017.02234)
Supplement: Supplementary file 7 [file Image_7.PDF]

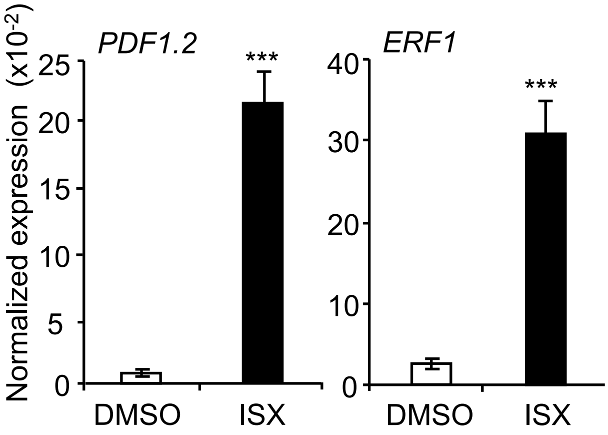

Fig. S7. **Expression of JA-regulated marker genes in wt seedlings treated or untreated with ISX 20 nM for 24h.** Expression of *PDF1.2*, and *ERF1* in wild-type seedlings treated with DMSO (mock) or ISX 20 nM for 24h. Analyses were performed by qRT-PCR and transcript levels are shown as the mean of three independent experiments (± SE; n=20 in each experiment) normalized to *PEX4* expression. Asterisks (***, P<0.001) indicate statistically significant differences according to Student's t test between ISX- and DMSO-treated samples.
